# Enrichment of sequencing targets from the human genome by solution hybridization

**DOI:** 10.1186/gb-2009-10-10-r116

**Published:** 2009-10-16

**Authors:** Ryan Tewhey, Masakazu Nakano, Xiaoyun Wang, Carlos Pabón-Peña, Barbara Novak, Angelica Giuffre, Eric Lin, Scott Happe, Doug N Roberts, Emily M LeProust, Eric J Topol, Olivier Harismendy, Kelly A Frazer

**Affiliations:** 1 Scripps Genomic Medicine, Scripps Translational Science Institute, The Scripps Research Institute, 3344 N. Torrey Pines Court, La Jolla, CA 92037, USA; 2 Division of Biological Sciences, University of California San Diego, 9500 Gilman Dr., La Jolla, CA 92093, USA; 3 Agilent Technologies, Inc., 5301 Stevens Creek Blvd., Santa Clara, CA 95051, USA; 4 Current address: Moores UCSD Cancer Center, 3855 Health Sciences Drive 0901, La Jolla, CA 92093-0901, USA

## Abstract

To exploit fully the potential of current sequencing technologies for population-based studies, one must enrich for loci from the human genome. Here we evaluate the hybridization-based approach by using oligonucleotide capture probes in solution to enrich for approximately 3.9 Mb of sequence target. We demonstrate that the tiling probe frequency is important for generating sequence data with high uniform coverage of targets. We obtained 93% sensitivity to detect SNPs, with a calling accuracy greater than 99%.

## Abstract 2

A method for target sequence enrichment from the human genome is described. This hybridization-based approach using oligonucleotide probes in solution has excellent sensitivity and accuracy for calling SNPs

## Background

Over the past several years, genome-wide association (GWA) studies have identified compelling statistical associations between more than 350 different loci in the human genome and common complex traits [1]. However, great difficulty occurs in moving beyond these statistical associations to identifying the causative variants and functional basis of the link between the genomic interval and the given complex trait. Population sequencing of these genomic intervals has been proposed as a method for identifying the causal common variants underlying the statistical associations and also for examining the potential contribution of rare variants in the interval to the complex trait of interest [1]. Next-generation sequencing technologies and their increased capacity have made it feasible to sequence efficiently hundreds of megabases of DNA. However, the current costs for sequencing entire human genomes makes this approach prohibitively expensive for population studies. Targeted sequencing of the specific loci associated with a complex trait in large numbers of individuals is a promising approach for using current sequencing technologies to identify and characterize the variants in these intervals. Additionally, population sequencing of candidate genes or the entire human exome may, in the near future, potentially make sequence-based association studies possible.

Several methods have been proposed for enrichment of sequence targets from the human genome. PCR has been used to amplify a large hundred-kilobase-size interval associated with prostate cancer for targeted sequencing in 79 individuals [2] and also the exons of hundreds of genes to identify somatic mutations in hundreds of individual tumors [3,4]. Although PCR enriches target sequences with high specificity and sensitivity, it is difficult to scale the method. A second approach is hybridization-based methods using oligonucleotide probes either attached to a solid array [5-7] or in solution [8] to capture the sequencing targets. The solid-phase hybridization approach has been used to capture the entire human exome, reported in several published studies [7,9]; however, the process is difficult to scale for large population studies. A proof-of-principle study for solution-phase hybridization by using long 170-bp capture probes has recently been published [8]. Although this study clearly demonstrated the utility of the approach, at a depth of 84× coverage, the variant-detection sensitivity was only 64% to 80% within the exonic sequences, likely because of insufficient coverage uniformity.

**Figure 1:**
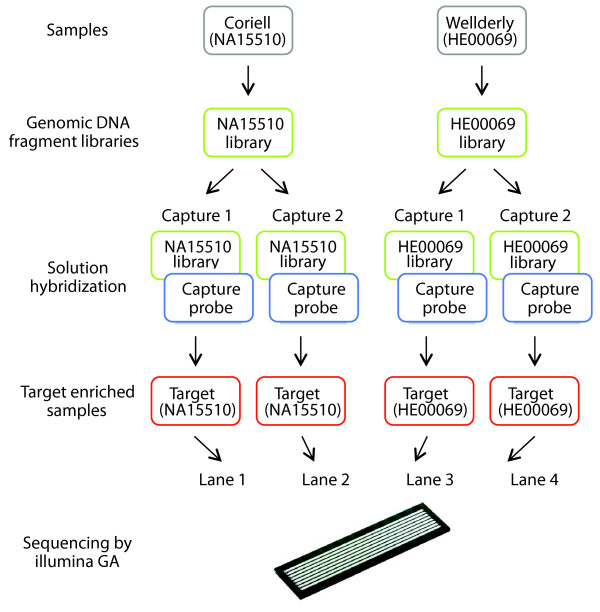
Experimental design. Genomic DNA fragment libraries were generated from two samples, Coriell (NA15510) and Wellderly (HE00069). Technical replicates of the target-enrichment steps for both samples NA15510 and HE00069 were performed (Capture 1 and Capture 2). The four target-enriched samples were loaded in separate lanes of a flow cell and sequenced by using the Illumina GAII.

In this study, we further assessed the solution hybridization method for enrichment of sequencing targets. We chose to sequence the exons and potential regulatory elements of 622 genes distributed across the genome that are candidate intervals for playing a role in healthy aging (Wellderly, denoting healthspan; Figure 1) [10]. These genes were selected either because their orthologues have been demonstrated to play a role in longevity in animal models [11-13] or for their potential roles in age-related diseases. We also included three contiguous genomic intervals on 8q24, 9p21, and 19q13, all of which contain variants associated with age-related diseases. Variants in the 8q24 interval have been associated with breast cancer [14], bladder cancer [15], and prostate cancer [16,17]. The 9p21 interval has been associated with coronary artery disease [18,19] and type 2 diabetes [20-22]. The 19q13 interval encodes the *APOE *gene, which is known to play an important role in Alzheimer disease [23,24] and coronary artery disease [25]. We prepared genomic DNA-fragment libraries with an average size of 200 bp from two samples, NA15510 and HE00069 (Figure 1). The fragment libraries for both samples were split into two aliquots, and technical replicates of the target-enrichment step (Capture 1 and Capture 2) were performed; each of the four target-enriched samples were loaded in separate lanes of an Illumina Genome Analyzer (GA) II flow cell (Illumina, San Diego CA, USA) and sequenced. To evaluate the approach, we analyzed the probe-design efficiency, the efficiency of capturing targeted sequences, coverage uniformity across targeted sequences, reproducibility for the technical replicates and across different samples, and single-nucleotide polymorphism (SNP) detection and accuracy rates. We demonstrated that the tiling frequency of the 120-bp capture probes is important for obtaining high uniform coverage across the targeted sequences; the reproducibility of coverage across samples is very good; and the resulting data have excellent sensitivity and accuracy for calling SNPs.

## Results and discussion

### Targeted genomic sequences

In total, about 3.6 Mb of human sequences consisting of three contiguous intervals (0.4 Mb) and the coding and potential regulatory elements of 622 genes (3.2 Mb) distributed across the genome were targeted for enrichment. The three contiguous genomic intervals spanned 125 kb on 8q24, 196 kb on 9p21, and 100 kb on 19q13 (Additional data file 1). The targeted sequences of the 622 genes comprised 9,215 exons and 4,886 evolutionarily conserved sequences (ECSs) located within 10 kb upstream or 20 kb downstream of the genes (Additional data file 2). ECSs were identified as stretches of contiguous sequence greater than 50 bases that had conservation scores of 0.75 or more within the 28-way placental mammalian conservation track at the UCSC genome browser [26].

### Probe design efficiency

**Figure 2:**
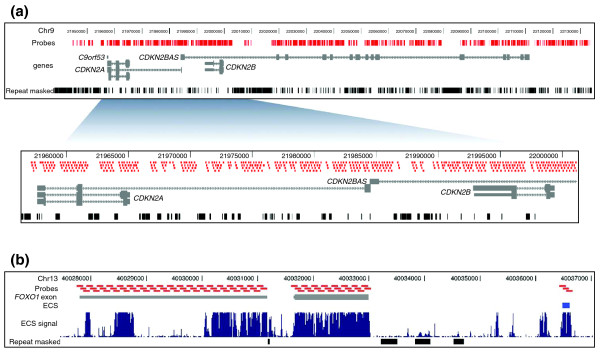
Distribution of capture probes. **(a) **The 196-kb targeted 9p21 genomic interval (positions 21,938,000-22,134,000, top panel) and a magnified view (positions 21,955,500 to 22,002,000) within the interval (bottom panel). The locations of the 120-mer capture probes designed by eArray are shown (*red bars*). Capture probes were designed to all sequences in the interval except for repetitive elements marked as Repeat masked (*black bars*). Exons (*grey rectangles*) and introns (*grey lines*) for genes in the interval are shown. **(b) **A 9-kb interval encoding the 3' UTR of the targeted *FOXO1*gene. Capture probes were designed to *FOXO1 *exons (*grey bars*) and ECS (*blue bars*), such as the one on the right end of the panel. The 2× probe tiling-frequency parameter results in adjacent 120-bp probes overlapping by 60 bp.

We submitted the sequences of the three contiguous genomic intervals and 622 genes to the web-based probe-design tool, eArray [27] for capture probe design. The repetitive sequences were masked by the RepeatMasker program (see Methods), and 120-mer capture probes were designed only for the unmasked unique sequences. As shown in Figure 2a, the repetitive elements in the contiguous genomic intervals are predominantly in introns and intergenic regions. The fraction of sequences for which capture probes could be designed in the 8q24, 9p21, and APOE intervals was 48.0% (60 kb of 125 kb), 55.1% (108 kb of 196 kb), and 37.0% (37 kb of 100 kb), respectively. Thus, the efficiency for designing capture probes for the three intervals varied substantially and reflects differences in the repetitive content of the intervals. Capture probes were successfully designed for 2.9 Mb (90.6%) of the 3.2 Mb of targeted sequences corresponding to the exons and ECSs of the 622 genes. The probe-design efficiency varied depending on whether the sequence was a coding exon (97%), a UTR (88%), or an ECS (86%). Because sequences encoding genes and ECSs are largely unique, the efficiency of designing capture probes for these elements is excellent. Multiple investigators have performed comparisons between RepeatMasker and masking by using 15-mer frequencies. These studies have shown that excluding sequences identified by the 15-mer frequency in the genome can result in fewer repetitive sequences being masked [5,28]. Therefore, a greater number of unique noncoding sequences could potentially be targeted if sequence masking were based on 15-mer frequencies. In the subsequent analyses, we considered only the targeted sequences for which capture probes have been successfully designed (approximately 3.9 Mb), which includes the 3.1 Mb of targets and the residual sequence of the 120 bp probe outside of the targeted coordinates.

### Efficiency of target enrichment

**Figure 3:**
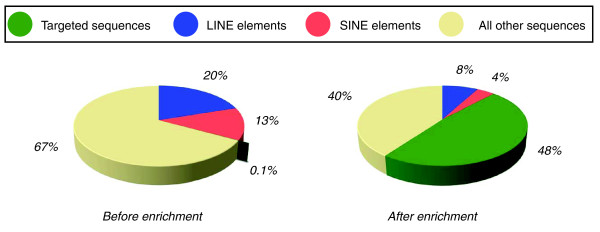
Efficiency of target enrichment. The pie chart on the left illustrates the relative percentages of the targeted sequences, LINE elements, SINE elements, and all "other" sequences in the human genome reference sequence (3.08 Gb in total). The pie chart on the right shows the relative percentages of these sequences in the filtered sequence reads (293 Mb in total). Targeted sequences include those on or near (target ± 150 bp) target.

To assess the efficiency, uniformity, and reproducibility of target enrichment by solution hybridization, we generated technical replicates of two samples. Specifically, we made genomic DNA-fragment libraries of two samples, NA15510 and HE00069, and performed the solution-hybridization step in replicate for each sample (Figure 1). We loaded the four target-enriched samples in separate lanes of an Illumina GAII flow cell (Illumina San Diego CA, USA) and sequenced by using 36-bp single reads. On average for each of the four samples, 12.4 million reads (446 Mb) came off the sequencer, of which 8.13 million reads (293 Mb) passed Illumina pipeline quality filters. Among the four lanes, no significant differences were found in the number of reads passing quality filters. Of the total reads, 76% (338 Mb/446 Mb) mapped uniquely to the genome, of which 43% (146 Mb of 338 Mb) mapped directly on the targeted sequences. Of the high-quality filtered reads, 87% (256 Mb of 293 Mb) mapped uniquely to the genome, of which 46% (135 Mb of 293 Mb) uniquely mapped directly on or near (target ± 150 bp) the targeted sequences, and 37% (109 of 293 Mb) uniquely mapped directly on the targeted sequences (Table 1 and Figure 3).

**Table 1:** Efficiency of target enrichment

Sample	Replicate	Filtered reads^1 ^(Mb)	Mapped bases, Mb					Uniquely mapped vases, Mb		
				
			HG18	On target	On or near target^2^	LINE	SINE	HG18	On target	On or near target^2^
NA15510	Capture 1	8,232,578 (296.4)	291.2	119.8	147.1	21.4	12.4	256.7	115.1	141.6
NA15510	Capture 2	8,106,056 (291.8)	286.8	107.1	130.9	24.1	13.9	252.8	102.9	126.0
HE00069	Capture 1	7,743,638 (278.8)	274.9	112.7	142.2	19.5	10.3	246.0	108.5	137.3
HE00069	Capture 2	8,451,260 (304.2)	300.3	112.1	140.2	24.9	12.8	268.5	108.0	135.4

	Average	8,133,383 (292.8)	288.3	112.9	140.1	22.4	12.4	256.0	108.6	135.1

^1^Number of high-quality reads from Illumina pipeline v1.3 and the megabases they compose.

^2^Near is defined as 150 bp upstream or downstream of the target sequence.

Repetitive elements compose a significant fraction of the human genome, and it is important to reduce their presence in the solution-hybridization step to enrich efficiently for targeted sequences. We examined the efficiency of masking repetitive elements during the capture probe design and of reducing their nonspecific hybridization by adding Cot-1 DNA in the solution-hybridization step by determining how many of the off-targeted sequences map to LINE and SINE elements that compose 20% and 13% of the human genome, respectively (Figure 3). Of the 52% (153 Mb) of filtered bases that do not map on or near target, 8% correspond to LINE, and 4%, to SINE elements, indicating that the fraction of sequences that are repetitive elements in the background of target enriched samples (Figure 1) is about one third of that in the genome at large. These data show that about 400-fold enrichment of the targeted sequences was achieved when capturing approximately 3.9 Mb by the solution-hybridization method.

### Uniformity of sequence coverage

**Figure 4:**
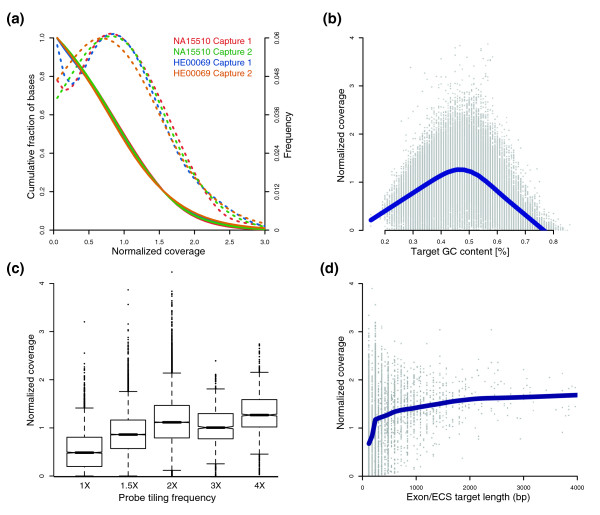
Uniformity of sequence coverage. Normalized coverage is the observed coverage of each base divided by the mean coverage of all the targeted bases to allow direct comparison among the four target-enriched samples. **(a) **Distribution of the normalized sequence coverage. The *solid lines *represent the cumulative fraction of bases (left axis) for each sample. The *dashed lines *(right axis) show a skewed normal distribution of the coverage for each sample. **(b) **A scatterplot of the normalized coverage of each capture probe versus the GC content of the probe. Normalized coverage was calculated by averaging across the four samples. **(c) **A box-whisker plot of the normalized coverage of capture probes versus tiling-probe frequency (see Methods). Each bin from 1× to 4× contains the data of 6,007, 15,513, 27,483, 1,132, and 3,184 probes, respectively. **(d) **A scatterplot of the normalized coverage versus the length of each targeted Exon/ECS; the x-axis is truncated because only a handful of exons are larger than 4 kb. The *solid blue lines *of **(c, d) **represent a polynomial regression of the scatterplot.

Uniformity of sequence coverage across targeted sequences is a key factor in determining the average depth to which samples have to be sequenced to cover underrepresented bases adequately. The four target-enriched samples had slightly different sequence yields (Table 1), and therefore, to allow a direct comparison of their coverage distribution, we normalized the coverage by dividing the observed coverage of each base by the mean coverage of all the targeted bases. For all four samples, the peak of the normal distribution curve is only slightly shifted to the left of the mean coverage (mean coverage, 29.1; normalized coverage, 1.0) (Figure 4a). On average, 91% of all the mapped bases fell between 1/5 and 5 times the mean coverage, and 98% were covered by at least one read (Table 2). Overall, our results demonstrate that the uniformity of sequence coverage across the targeted sequences is excellent. Furthermore, our results suggest that studies using solution hybridization to enrich for 3.9 Mb of targeted sequences and the Illumina GA platform aiming for about 12 million 36-bp reads per sample will result in greater than 88% of the bases having seven or more reads.

**Table 2:** Uniformity of sequence coverage

Sample	Replicate	Mean coverage	Proportion of mapped bases on targets^1 ^(%)			
			
			>1 read	<1/5	1/5 to <5	>5
NA15510	Capture 1	30.8	98.24	10.27	89.71	0.02
NA15510	Capture 2	27.6	98.34	9.34	90.64	0.02
HE00069	Capture 1	29.0	98.24	8.98	91.00	0.02
HE00069	Capture 2	28.8	98.40	8.48	91.51	0.02

	Average	29.1	98.31	9.27	90.72	0.02

^1^Percentage of all bases that had more than one read or had a sequence depth of less than 1/5 the mean coverage, between 1/5 and 5 times the mean, and greater than 5 times the mean.

We expect capture probes of different GC content to behave differently in the solution-hybridization step and that this would have an effect on the resulting sequence coverage. We plotted the GC content of each probe versus the normalized coverage of the probe (Figure 4b). The GC content of the capture probes ranged from 15% to 86%, and, as expected, the scatterplot appears to have a gaussian distribution, with the peak of the distribution at about 45% GC content. The normalized coverage decreased to less than 0.5 when the GC content was lower than about 23% or higher than about 66%. These results seem to be reflecting the low efficiency of hybridization to the targets with base composition of either AT or GC rich. However, other possible explanations exist for these observations, including potential oligonucleotides synthesis issues of high- and low-GC content probes, potential self-structure of targeted DNA during hybridization, and potential biases in the PCR amplification step during generation of the sequencing libraries, resulting in fewer corresponding targeted sequences [29].

### Effect of probe-tiling frequency on sequence coverage

To gain insight into the optimal density for tiling the capture probes, we assessed the effect of probe-tiling frequency on sequence coverage (Figure 4c). As described in Methods, the probe-tiling frequency varied from 1× to 4× for the targeted sequences. We separated the 52,187 capture probes into five bins based on their probe-tiling frequency then plotted the distribution of the normalized coverage for each bin (Figure 4c). The normalized coverage increased from 1× to 1.5× to 2× probe-tiling frequency and then formed a plateau. These results suggest that sequence coverage is improved if each targeted base pair is contained within two different capture probes but is not affected by a greater tiling density. To examine further the effects of probe-tiling frequency, we plotted the length of targeted exons and ECS regions compared with normalized coverage (Figure 4d). The lengths of the targets varied from 120 bp to 7,860 bp, and targets less than 180 bp in length (1 through 1.5× tiling frequency) had less coverage than longer exonic sequences, which had 2× tiling frequency (see Methods). These results indicate that, for optimal coverage of human exons shorter than 180 bp in length [30], at least three 120-mer capture probes per exon should be used to achieve an optimal tiling frequency.

### Reproducibility

The ability to capture reproducibly targeted sequences across multiple samples is of high importance to perform sequence-based association studies. We assessed the reproducibility of this enrichment method by comparing technical replicates of the solution-hybridization step (Figure 1). For each of the two samples, a single genomic DNA library was generated, and two aliquots of each sample library were independently hybridized with capture probes (Capture 1 and Capture 2).

**Figure 5:**
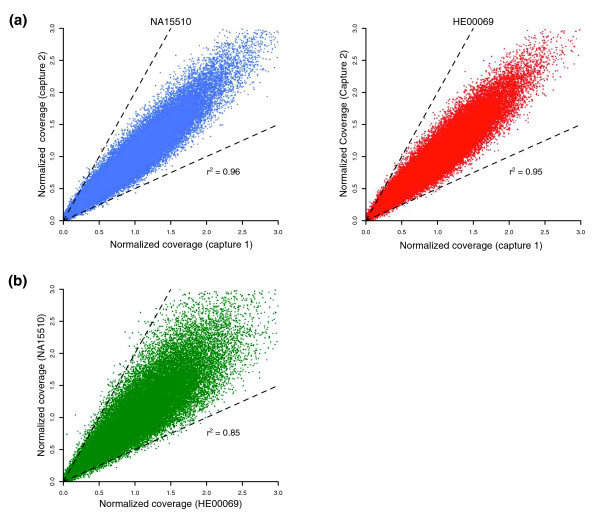
Reproducibility of target enrichment. **(a) **The normalized mean coverage of each capture probe is plotted for the technical replicates of NA15510 and HE00069 (Capture 1 versus Capture 2). Capture probes that lie outside the *dashed lines *on the plot have normalized coverage that differs by more than twofold in the technical replicates. **(b) **The normalized mean coverage of each capture probe is plotted for NA15510 (Capture 1) versus HE00069 (Capture 1). The values of coefficient of determination (*r*^2^) are shown.

To test truly the reproducibility of the solution-hybridization step, we had two different individuals at two different sites perform the technical replicates. We first compared the normalized coverage of each capture probe between the technical replicates of the same sample (Figure 5a). The results show that the reproducibility between Capture 1 and Capture 2 was excellent, with high correlation within the same sample (*r*^2 ^= 0.96 for NA15510, and *r*^2 ^= 0.95 for HE00069).

We next examined sample-to-sample reproducibility by comparing the normalized coverage of one technical replicate of NA15510 with one technical replicate of HE00069 (Figure 5b). The correlation of the two samples was very good (*r*^2 ^= 0.85) but significantly lower than that observed for the technical replicates of the same sample. These results could reflect sequence-variant differences in the two samples or that differences in the genomic DNA-fragment library step may affect the solution-hybridization step, or both. In either case, our results suggest that the reproducibility of capturing targeted sequences across samples in different experiments will be sufficient to allow sequence-based association studies.

### Accuracy of variant calling

**Figure 6:**
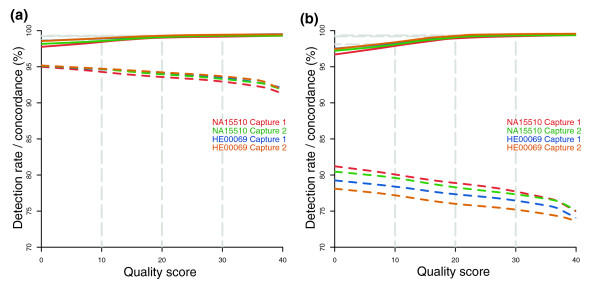
Accuracy of sequence variant calls compared with microarray genotype calls. The detection rate (*dashed lines*) and concordance (*solid lines*) of variant calls versus the MAQ quality score [33] are shown for target sequences **(a) **and on or near (target ± 150 bp) targets **(b)**. A filter requiring five or more reads was first applied, and then the detection and concordance rates at the various MAQ quality score thresholds was determined.

We evaluated the accuracy of the variant bases called in the targeted sequences for the two samples by comparison with Illumina 1 M microarray genotypes (Figure 6 and Table 3). About 4,050 SNPs found within the targeted sequence were surveyed on the microarray. We were able to call confidently (MAQ consensus score was 30 or more and at least 5× coverage) 93.4% of these SNPs, with an accuracy rate of 99.7% (Figure 6a, Table 3). We then assessed our ability to call variants outside of the defined target region. Of the approximately 6,700 variants, on or near target (±150 bp), which includes the approximately 4,050 on-target variants, we were able to call confidently 76.7% of the SNPs with an accuracy rate of 99.6% (Figure 6b, Table 3). These results indicate that the sensitivity and accuracy of identifying SNPs in the targets are excellent and that variants in the sequences that lie immediately outside the targets can be identified, albeit with a lower detection rate.

**Table 3:** Variant detection rate and concordance^1^

		Microarray SNPs^2^		Variant detection rate (%)		Variant concordance^3 ^(%)		Number of discordant SNPs	
					
Sample	Replicate	On target	On or near target	On target	On or near target	On target	On or near target	On target	On or near target
NA15510	Capture 1	4,062	6,682	93.5	76.4	99.7	99.6	10	20
NA15510	Capture 2	4,062	6,682	93.7	75.2	99.8	99.8	8	10
HE00069	Capture 1	4,055	6,675	93.0	77.7	99.5	99.5	19	27
HE00069	Capture 2	4,055	6,675	93.4	77.4	99.6	99.6	14	22

	Average	4,059	6,679	93.4	76.7	99.7	99.6	13	20

^1^Only variant bases with an MAQ quality score of 30 or greater are included in this table.

^2^Number of microarray genotypes in the on-target sequences or in the on or near (target ± 150 bp) target sequences.

^3^Number of genotypes that match between sequence and microarray genotype divided by total comparisons.

To gain insight into the source of the variant-calling errors in the sequence data, we carefully examined the discrepancies that occurred for on-target bases with five reads and an MAQ quality score of 30 or more. In the four samples, in total, 51 discrepant variants were found across 33 positions (Additional data file 3). For 24 of the discrepancies, the variant calls between the two replicates agreed with each other, suggesting that the microarray data are incorrectly calling the SNP, but not ruling out the less-likely possibility of a systematic error in the sequencing. In 18 of the discrepancies, the replicate sample was unable to make a high-quality call. The majority of these positions (72%) were missed heterozygote genotypes in which the sequence coverage was low for both samples. The remaining nine discrepancies were called correctly in one of the replicates and incorrectly in the second. All but one of the variants were below the mean coverage, and the majority (six of nine) were missed heterozygote calls. It is important to note that these errors represent a minor fraction of the heterozygous sites and that the vast majority are correctly called in the sequence data. Twenty-two positions were discordant in only one replicate, the majority of which (64%) failed to be called in the second replicate. The remaining one position was discordant in one NA15510 and one HE00069 replicate and not called in the other replicate. These results suggest that approximately half of the discrepant variants bases are likely attributable to errors in the microarray data, and the other half are likely errors in the sequence data. Thus, the accuracy of calling SNPs in the sequence data may be greater than 99.7% (Table 3). Additionally, because most of the discrepancies attributed to sequencing errors were of lower coverage, it is reasonable to assume that an increase in sequencing depth or capture uniformity would rescue these variants.

### Novel and functional variants

Ascertaining base calls at known variant loci (dbSNP) gives only a partial and abstract view of variant calling. To interpret our sequencing data from a more practical angle, applicable for exons-sequencing studies, we considered all variants (including novel variants not in dbSNP) found in our study and predicted their impact on protein function. To increase our confidence in the calls at novel variant loci, we combined the two replicates and kept only concordant calls between them. Of the 1,049 exonic variants detected in HE00069, 75 (7.1%) were novel variants; this fraction was slightly lower for NA15510, in which 51 (5.1%) of the 1,002 variants were novel. This lower percentage is likely because NA15510 is a publicly available resource that has contributed to the discovery of SNPs found in dbSNP, thus causing an ascertainment bias for this particular sample. Consistent with previous reports [9,31], the majority of the novel variants are heterozygous (Table 4).

**Table 4:** Zygosity and functional annotation of exonic variants

Variant type	NA15510				HE00069			
		
	Heterozygote		Homozygote		Heterozygote		Homozygote	
				
	All	Novel	All	Novel	All	Novel	All	Novel
Exonic	699	50	303	1	719	67	330	8
--- coding	356	18	165	0	359	19	157	3
--- 3' UTR	306	12	120	1	325	24	158	2
--- 5' UTR	37	1	18	0	35	4	15	0
Nonsynonymous	129	8	62	0	140	11	65	2
Synonymous	227	10	103	0	219	8	92	1
Protein damaging	5	1	4	0	8	1	5	0

Both samples had roughly an equal number of nonsynonymous variants in the 622 genes, with one in every five genes having a heterozygote and one in every 10 having a homozygote nonsynonymous variant. Most of the nonsynonymous SNPs are present in dbSNP and might thus be common variants not specific to our samples (Table 4). Of the 191 nonsynonymous variants found in NA15510, nine were predicted to cause an amino acid substitution that results in a functional change, as determined by the program SIFT [32]. This number was slightly higher in HE00069, with 13 of the 205 nonsynonymous changes predicted to cause a change in function.

Only a few extensive coding variation surveys have been performed in the human genome. Our analysis is consistent with previous whole-exome analysis [33] and thus supports the use of solution hybridization for targeted exon sequencing.

## Conclusions

Our results show that the solution hybridization-based method can generate highly uniform coverage of sequence targets that is reproducible across samples. The method has limited, if any, systematic allelic biases resulting in dropout effects, as demonstrated by the greater than 99% SNP calling accuracy and especially the ability to call correctly most heterozygous sites. The solution hybridization-based method is clearly dependent on the ability to design successful capture probes to target sequences of interest. The ability to design capture probes is dependent on local sequence characteristics, and whereas 97% of the base pairs in exonic targets can be targeted, the success rate is only about 50% for base pairs in genomic intervals. We demonstrated that shorter 120-mer probes and an overlapping tiling strategy for probe design produces greater uniformity than previously published results for a solution hybridization-based study with 170-mer probes tiled with an end-to-end strategy [8]. It is important to note that some of this increase in overall uniformity of coverage may in part be due to the fact that the shorter 120-mer probes are easier to synthesize in a reliable and consistent fashion than are 170-mer probes. This greater coverage uniformity allowed us to call confidently a higher proportion of variant bases at a sequence-coverage depth almost one third lower than that produced in the previous study. This improvement will result in reduced costs and more-complete variant detection for large-scale resequencing studies.

Two general types of population-based sequencing studies are currently under consideration in the community. The first type is sequence-based association studies that specifically focus on elements with known function. Relatively few repetitive sequences occur in the majority of known functional elements, and thus the success rate for designing capture probes is high. The second type is targeted sequencing of intervals associated through genome-wide association studies with a particular complex trait. In our study, the repetitive content of the three genomic intervals we targeted varied from 45% to 63%. Thus, although the base pairs in these genomic intervals for which capture probes can be designed are well represented in the resulting sequence data, a considerable fraction of bases cannot be investigated. It is important to note that analysis methods for investigating variants outside of exons and regulatory elements for function are currently nonexistent. Thus, the solution-hybridization approach for targeted sequencing is clearly optimal for sequence-based association studies, and the limitations of capture-probe design have to be taken into account for targeted sequencing of genomic intervals.

Overall, our study demonstrates that the solution hybridization-based method is well suited for the enrichment of loci in the mega-base-pair scale from the human genome for population studies using current sequencing technologies.

## Materials and methods

### Genomic DNA

One sample (NA15510; Caucasian) was obtained from the Coriell Institute for Medical Research [34], and the second sample (HE00069; Caucasian) was obtained from the Scripps Translational Science Institute [35] "Wellderly" cohort. The genomic DNA for NA15510 was isolated from Epstein-Barr virus-transformed cell line. The Wellderly Study has been approved by Institutional Review Board of Scripps Health, and enrollment of participants and blood collection were carried out in accordance with the Helsinki Declaration. Genomic DNA of HE00069 was isolated from blood by the PAXgene Blood DNA Kit (Qiagen, Inc., Valencia, CA, USA), according to the manufacturer's instructions.

### Probe design and synthesis

The biotinylated-cRNA probe solution was manufactured by Agilent Technologies and was provided as capture probes. The sequences corresponding to the three genomic intervals and the 622 genes were uploaded to the Web-based probe-design tool, eArray [27]. The coordinates of the sequence data in this study are based on NCBI Build 36.1 (UCSC hg18). The following parameters chosen were capture-probe length (120 bp), capture-probe tiling frequency (2×), allow overlap into avoid regions (20 bp), and avoid standard repeat masked regions option (eliminates repetitive sequences by using the RepeatMasker program alignment-based method). The 2× tiling-frequency parameter designed one capture probe for targeted sequences 120-bp or more (1× coverage per capture probe), two probes for targeted sequences between 120 and 180 bp (1.5× coverage per capture probe), and base pairs in targeted sequences more than 180 bp have 2× coverage, except for those at the ends of the sequence, which are covered at 1.5×. The genes in the targeted intervals also were included individually in the set of 622 genes. Therefore, the probe-tiling frequency varied from 1× to 4× for the targeted sequences. In total, 52,187 probes were designed (Additional data file 4), synthesized on a wafer, subsequently released off the solid support by selective chemical reaction, PCR amplified through universal primers attached on the probes, and then amplified and biotin-conjugated by *in vitro *transcription [8].

### Genomic DNA-fragment library

Genomic DNA-fragment libraries were prepared according to the manufacturer's instructions (Illumina, Inc., San Diego, CA, USA) with slight modifications, as described [29]. In brief, 3 μg of each genomic DNA (NA15510 and HE00069) was fragmented by Adaptive Focused Acoustics (Covaris S2; Covaris, Inc., Woburn, MA, USA) by using the following conditions: 20% duty cycle at intensity 5 for 90 seconds with 200 cycles per burst. This resulted in fragmentation of the genomic DNA to an average size of about 200 bp. After end repair and A-base tailing, the Illumina single-end adaptor was ligated. After size selection for a mean insert size of about 250 bp, each fragment library was enriched by 14-cycle PCR amplification by using 4 μl per fragment library as a template. The PCR-amplified fragment libraries were quantified by NanoDrop (ND8000; NanoDrop Technologies, Inc., Wilmington, DE, USA).

### Solution hybridization and target enrichment

Technical replicates of the target-enrichment step for both samples NA15510- and HE00069- were performed (Figure 1). The genomic DNA-fragment libraries of the samples were split into two aliquots, with the target-enrichment step performed on one aliquot at Agilent Technologies and on the other aliquot at the Scripps Translational Science Institute. At both institutes, the same protocol was used from the solution hybridization through the PCR-enrichment steps. In a PCR plate, one unit of the capture probe (Agilent Technologies, Inc., Santa Clara, CA, USA; ELID number: 0220261) was mixed with 20 units of RNase inhibitor (SUPERase-In, Ambion, Inc., Austin, TX, USA), heated for 2 min at 65°C in GeneAmp PCR System 9700 thermocycler (Applied Biosystems, Inc., Foster City, CA, USA), and then mixed with prewarmed (65°C) 2× hybridization buffer (Agilent Technologies, Inc., Santa Clara, CA, USA; part number: G3360A). In a separate PCR plate, 500 ng of each genomic DNA-fragment library was mixed with 2.5 μg of human Cot-1 DNA, 2.5 μg of salmon sperm DNA, and 1 unit of blocking oligonucleotides complementary to the Illumina single-end adaptor, heated for 5 minutes at 95°C, and held for 5 minutes at 65°C in the thermocycler. Within 5 minutes, the mixture was added to the capture probes, and the solution hybridization was performed for 24 hours at 65°C.

After the hybridization, the captured targets were selected by pulling down the biotinylated probe/target hybrids by using streptavidin-coated magnetic beads (Dynal DynaMag-2; Invitrogen Corporation, Carlsbad, CA, USA). The magnetic beads were prepared by washing 3 times and resuspending in binding buffer (1 *M *NaCl, 1 m*M *EDTA, and 10 m*M *Tris-HCl, pH 7.5). The captured target solution was then added to the beads and rotated for 30 minutes at room temperature. The beads/captured targets were then pulled down by using a magnetic separator (DynaMag-Spin; Invitrogen Corporation), removing the supernatant, resuspending in prewarmed (65°C) wash buffer (Agilent Technologies, Inc.; part number: G3360A), and then incubated for 15 minutes at room temperature. The beads/captured probes were then pulled down with the magnetic separator and washed by resuspension and incubation for 10 minutes at 65°C in wash buffer. After three washes, elution buffer (0.1 *M *NaOH) was added and incubated for 10 minutes at room temperature. The eluted captured targets were then transferred to a tube containing neutralization buffer (1 *M *Tris-HCl, pH 7.5) and desalted with the MinElute PCR Purification Kit (Qiagen, Inc., Valencia, CA, USA). Finally, the targets were enriched by 18-cycle PCR amplification by using 1 μl per sample as a template, and the amplified targets were purified by QIAquick PCR Purification Kit (Qiagen, Inc.).

### Sequencing by Illumina GAII

The four target-enriched samples (Figure 1) were quantified by PicoGreen dsDNA Quantitation Assay (Invitrogen Corporation) in quadruplicate. The samples were diluted to 10 n*M*, denatured with NaOH, and then 2.3 p*M *of each target-enriched sample was loaded into separate lanes (lane 1 to lane 4) of the same flow cell. Sequencing was performed for 36 cycles by using Illumina Single-Read Cluster Generation Kit and 36 Cycle Sequencing Kit according to manufacturer's instructions.

### Mapping, coverage uniformity, and SNP detection

The sequencing data produced by Illumina GAII were processed through the Illumina pipeline v1.3 by using default parameters. For all analyses, the high-quality filtered reads were mapped to the reference sequence (NCBI Build 36.1, UCSC hg18), by using MAQ v0.71 [36] default parameters, except for the allowing of three mismatches during alignment (-n 3). SNP calling was performed by using the Perl-based SNP filter of MAQ after alignment (-map), assembly (-assemble), and consensus calling (-cns2snp). We used the default parameters for both SNP calling and alignment, with the exception of variant quality score of 30 or more and a read depth of 5 or greater. Variants with less than five reads or a quality of less than 30 were marked as no calls. Sequence variants were compared with microarray genotypes generated for both samples (NA15510 and HE00069) by using the Illumina 1 M Infinium bead arrays according to manufacturer's instructions. Illumina 1 M genotypes were converted to reference strand positive from dbSNP forward Bead Studio reports. We removed 15 SNPs from the reports because of discrepancies between Illumina genotypes and dbSNPs reported strands and alleles. Calls were considered discordant regardless of the type of discordance; for example, an AB to AA error affected the concordance score the same as an AA to BB error. All coverage calculations were performed with a combination of custom Perl scripts and the statistics package R. Coverage and uniformity calculations were performed by using the 3.9 Mb of targeted sequence. Mean coverage was calculated by total bases on target divided by 3,886,910 (total bases captured). Normalized coverage for each base was calculated by dividing the coverage at that base by the mean coverage for the sample. For functional analysis of the variants, the variants in the two replicates were combined, and only those with matching high-quality calls in both samples were considered for analysis. Variants were processed through the SIFT program [32] to determine their functional role.

## Abbreviations

ECS: evolutionarily conserved sequence; GWA: genome-wide association; SNP: single-nucleotide polymorphism.

## Authors' contributions

OH, DNR, and KAF designed the study. BN designed probes. AG and SH prepared capture probes. MN, XW, CPP, and EL performed experiments. MN and RT analyzed the data. EJT, EML, and KAF supervised the work. MN, RT, and KAF wrote the manuscript. All authors read and approved the final manuscript.

## Additional data files

The following additional data are available with the online version of this article: an Excel file listing the three contiguous genomic intervals that we have targeted in this study (Additional data file 1), an Excel file listing the genes and ECS that we have targeted in this study (Additional data file 2), the 33 discordant positions and the variant characteristics in each targeted sequencing experiment (Additional data file 3), and an Excel file listing the probes that we have designed by using eArray[27]) in this study (Additional data file 4).

## Supplementary Material

Additional data file 1 Contiguous genomic intervals targeted in the present study Click here for file

Additional data file 2 Genes and ECS targeted in the present study Click here for file

Additional data file 3 Discordant positions and the variant characteristics in each targeting sequencing experiment Click here for file

Additional data file 3 Probes designed using eArray Click here for file
